# Personal

**Published:** 1895-11

**Authors:** 


### Personal.

MEDICAL Director Albert Leary Gihon, United States Navy, the senior officer of the medical corps of the navy, who received retirement orders September 28, 1895, is one of the most eminent men of his profession in the navy. He has a fine service record of forty years and leaves the navy under the age-retiring law. He received his degree of A. B. at the age of seventeen, M. D. two years later, and at the age of twenty-one was professor of chemistry and toxicology in the Philadelphia College of Medicine and Surgery. He has served in all parts of the world in the navy and has received decorations and thanks from several foreign countries for services rendered.

He is the author of numerous papers and addresses on naval hygiene, public health, sanitary reform, state medicine, higher medical education, vital statistics, medical demography and climatology, and has contributed to literary magazines and other periodicals articles on medical and surgical subjects. One of his latest contributions to medical science was alluded to in the review of the Twentieth Century Practice in the September issue of this journal, page 195.

Dr. Gihon is originator of the project to erect a monument at Washington to Dr. Benjamin Rush. Dr. Gihon is in unimpaired physical and mental vigor, a fact that promises continued usefulness as one of the foremost medical men of the century. He retires with the rank of commodore, U. S. Navy, which corresponds to that of brigadier-general in the army.

---

Dr. Dewitt G. Wilcox, of 568 Delaware avenue, Buffalo, announces his retirement from general practice to devote himself exclusively to surgery, including surgical gynecology. His relations to the Lexington Heights Hospital will continue as heretofore.

---

Dr. J. L. Eddy, of Olean, was elected president of the Erie Railway Surgeons' Association at the annual meeting held in Buffalo, October 1, 1895. Dr. Eddy is one of the well-known surgeons of the Southern tier.

---

Dr. Ernest L. Ruffner, whose term of service as one of the resident staff of the Buffalo General Hospital terminated May 1, 1895, has become associated with Dr. Floyd S. Crego, 469 Delaware avenue.

---

Dr. C. R. Holmes, of Cincinnati, announces his removal to his new offices and private hospital at Nos. 8 and 10 East Eighth street, where only ophthalmic, aural and rhinological cases will be admitted.

